# A randomized controlled trial of group psychological intervention combined with medication for treating adolescent depression

**DOI:** 10.3389/fpsyg.2025.1657172

**Published:** 2025-09-15

**Authors:** Xinming Yu, Zijun Chen, Bin Wang, Ke Zheng, Yong Liang, Jian Guo, Shikang Li, Liang Yu

**Affiliations:** ^1^The Second People’s Hospital of Chuzhou City, Chuzhou, China; ^2^Wannan Medical College, Wuhu, China

**Keywords:** group psychotherapy, adolescent, depression, HAMA, HAMD-17

## Abstract

**Objective:**

A study of the effects of group psychotherapy on depression and anxiety in adolescents.

**Methods:**

Ninety-two adolescent depression patients attending a city mental health center from May 2022 to December 2024 were selected for the study, and a randomized controlled trial design was used with 46 cases in each group. The patients in the observation group were treated with group psychotherapy combined with medication. The patients in the control group were treated with medication, for four consecutive weeks, and *t*-tests and chi-square tests were used to compare the differences in the rates of HAMA and HAMD-17 score reduction between the two groups.

**Results:**

(1) The difference in HAMD-17 scores between the two groups at baseline was not statistically significant (*Z* = −0.455, *p* = 0.649). In contrast, the difference in HAMA scores between the two groups at baseline was statistically significant (*Z* = −2.120, *p* = 0.034). Four patients in each of the two groups dropped out of the treatment, resulting in a total dropout rate of 8%. (2) At the end of the 4-week treatment, the HAMA and HAMD-17 scores of patients in the two groups were significantly lower than those at baseline, and the difference in the rate of reduction in HAMA and HAMD-17 scores before and after the treatment between the observation group and the control group (89.1% vs. 34.8, and 97.8% vs. 80.4%) was statistically significant (*χ*^2^ = 28.82, *p* < 0.001; *χ*^2^ = 7.18, *p* = 0.007), indicating clinically meaningful improvement as both rates far exceed the 50% threshold for treatment response.

**Conclusion:**

After group psychotherapy combined with medication to treat depression symptoms in adolescents is more effective, it can effectively accelerate the recovery of the disease, improve negative emotions, and reduce the recurrence of the disease.

## 1 Introduction

In recent years, the prevalence of adolescent depression has shown a significant increase, and the prevalence also shows a tendency of increasingly younger age of onset (Chen and Wang, 2013; Rawana and Morgan, 2013; Zhou and Jiang, 2024). The main symptoms of adolescent depression are reduced interest, difficulty in concentration, excessive self-blame, and negative emotions such as sadness, anxiety, and despair (Ma et al., 2019; Mendelson and Tandon, 2016). These symptoms not only impair adolescents’ cognitive abilities and executive functioning but may also lead to severe impairment of social function (Zhong et al., 2023). Adolescents with more severe symptoms are at higher risk for future functional and psychological impairments, which can have a significant negative impact on their growth and development.

However, compared to adults, depression in adolescents is often overlooked and is not easily detected, diagnosed, or treated (Yang and Song, 2025; Zhang, 2007), and its development process is also more gradual and enduring. Many patients are already experiencing significant impairment of social functioning, accompanied by a very high risk of suicidal ideation and suicidal behavior (Li and Zhang, 2023), by the time they seek medical help (Luo et al., 2008). Therefore, it is particularly critical to develop and implement comprehensive and effective treatment programmes for children and adolescents with depressive disorders as early as possible.

Due to the lack of experimental data on the safety of medications for children and adolescents, the range of medications available for antidepressant treatment in adolescents is relatively limited. This dilemma makes it even more difficult to alleviate adolescent depressive symptoms, and it is difficult to adequately meet the actual needs of treatment. Therefore, the study of combined group psychotherapy and pharmacological treatments for adolescent depression is a pressing issue in the current period.

Irvin D. Yalom (Yalom and Leszcz, 2010), a master of group psychotherapy, believes that the problems between people are more important than the problems within people, and that group psychotherapy is a form of psychological counseling carried out in a group situation, which, through the interaction of interpersonal relationships within a group, prompts individuals to learn, experience, know themselves, accept themselves, adjust and improve their relationships with others, learn new attitudes and behaviors to develop good adaptability to society, and help others. It is a process of helping others by improving relationships with others, learning new attitudes and behaviors, and developing the ability to adapt to society. We have explored the use of group psychotherapy and medication based on humanistic theory, kinetic theory, cognitive behavioral theory and family therapy theory system for adolescent depression patients, and conducted empirical studies to improve the control of adolescent depression, the recovery cycle and the probability of recurrence of the disease, which has achieved good results.

The 11 therapeutic factors proposed by Aron are particularly applicable to adolescents, as their development focuses on identity formation, peer recognition and social belonging. Adolescence is characterised by increased sensitivity to interpersonal, isolation and existential issues. Factors such as universality, interpersonal learning and group cohesion directly counteract the core vulnerability of adolescent depression. These elements align with adolescents’ need for peer connection while providing a structured space to practice social skills and self-disclosure, critical for developmental milestones compromised by depression (Fletcher, 2008).

## 2 Objects and methods

### 2.1 Objects

A total of 92 patients with depression who visited this mental health center from May 2022 to December 2024 were selected as the study subjects. They were divided into an observation group (46 cases) and a control group (46 cases) using a randomized controlled grouping method. The experimental group ranged in age from 12 to 16 years, with an average age of (14.65 ± 1.22) years. The control group also ranged in age from 12 to 16 years, with an average age of (14.17 ± 1.14) years (Table 1). This study was reviewed and approved by the Ethics Committee of a municipal mental health center, and permission was granted to conduct this research.

**Table 1:** General demographic data.

Project	Control group (*n* = 46)	Observation group (*n* = 46)	*χ*^2^-value/*t*-value	*p*-value
Age	14.17 ± 1.14	14.65 ± 1.22	1.946	0.055
Gender				
Male	9	11	0.064	0.800
Female	37	35		
Level of education				
Primary school	2	1	5.720	0.126
Junior high school	29	20		
High school	12	23		
Vocational high school	3	2		
Family situation				
Parents	37	34	2.810	0.422
Single parent	7	9		
Blended family	2	3		
Only child				
Yes	15	18	0.189	0.664
No	31	28		

Inclusion criteria for patients: Patients admitted to the Children and Adolescent Department of our center between May 2022 and December 2024; aged between 12 and 18 years; diagnosed according to the diagnostic criteria of the International Classification of Diseases, Tenth Revision (ICD-10) through three-level ward rounds during hospitalization; good patient compliance, mild-to-moderate depressive symptoms at enrollment, relatively stable condition, certain communication abilities, mild impairment of social functioning, and intact insight; HAMD-17 total score ≤17 and >7; disease duration >2 weeks; normal intelligence. (Good patient compliance: Defined as ≥80% adherence to medication and attendance at ≥90% of scheduled clinical appointments in the past month; Relatively stable condition: No hospitalization for acute exacerbation in the past 4 weeks, absence of suicidal ideation (Columbia-Suicide Severity Rating Scale score ≤2), and no severe agitation or psychosis.)

Exclusion criteria for patients: Presence of severe diseases in vital organs such as the heart, liver, or kidneys; history of severe organic brain disorders or addiction to psychoactive substances; the presence of other severe mental illnesses; history of individual psychotherapy within the 3 months before treatment; severe suicidal tendencies or behaviors; patients or their families who are resistant to treatment, or those who miss sessions without reason or withdraw midway.

### 2.2 Sample size

The researchers anticipated a significant difference in the reduction rates of HAMD-17 and HAMA scores between the observation group and the control group. Previous studies and pilot data informed the expected effect size, ensuring that the sample size would be sufficient to detect clinically meaningful differences. To achieve adequate statistical power and a significance level of 0.05, the researchers calculated the required sample size. This ensured that the study would have a high probability of detecting true effects if they existed. The study employed a randomized controlled trial (RCT) design, with equal allocation to the observation and control groups. The initial target was 50 participants per group to account for potential dropouts and ensure balanced group sizes. Anticipating a dropout rate of approximately 8%, the researchers initially planned for 50 participants per group. After excluding four dropouts from each group, the final sample size was 46 participants per group, totaling 92 participants. This adjustment maintained the study’s power and validity.

### 2.3 Method

#### 2.3.1 Randomized group

This study is an open randomized controlled trial. Adolescent depressive patients receive routine pharmacological treatment upon admission and are simultaneously randomized into groups. Randomization was conducted based on computer-generated random number sequences. It is anticipated that 50 cases will be collected for the pharmacological treatment group (control group) and the combined group psychotherapy and pharmacological treatment group (observation group), respectively. After excluding four lost-to-follow-up cases from each group, 46 cases will be included in each group. A SPSS was used to generate the random allocation sequence. The randomization was 1:1. Each enrolled participant was assigned a unique ID number. The software uses a pseudorandom algorithm to determine group allocation, “1” = observation group, “2” = control group. Given the behavioral nature of group psychotherapy, neither participants nor therapists were blinded to allocation.

#### 2.3.2 Treatment

(1) Observation Group: Each group consists of 10 patients and two therapists, forming a closed, structured group. The treatment lasts for 4 weeks, with two sessions per week, each lasting 80 min. The psychotherapists in the observation group all hold nationally registered psychotherapist qualifications and have received systematic training in group psychotherapy. They are supervised during the treatment period. The format of the group psychotherapy is arranged as follows: a total of 11 factors, conducted over eight sessions. The members randomized into the observation group are divided into five groups, with 10 individuals forming one group, totaling five groups formed sequentially. The group leaders for all five groups are experienced psychotherapists who have also undergone professional training in the theory and practice of this group counseling. Each group arranges a family member interview session before the second-to-last group therapy session. Health education and related knowledge dissemination are provided to the primary family members of the patients. This aims to moderately improve the family living environment of the patients and prepare for consolidating the effects of group therapy after the patients return home.

Group psychotherapy is a highly effective psychological treatment method, offering many advantages that individual psychotherapy does not provide. Additionally, group psychotherapy can accommodate more patients and generate significant economic benefits (Beck and Coffey, 2005). By integrating the 11 therapeutic factors proposed by Irvin D. Yalom and systematically organizing the goals and content of these factors (Table 2), therapists can design intervention strategies in a more structured manner, thereby enhancing the effectiveness of group therapy.

**Table 2:** 11 therapeutic factors.

Efficacy factors	Therapeutic target	Structured treatment content
Instillation of hope	Inspire members’ confidence in change and recovery, reducing feelings of helplessness. 2. Help members recognize the effectiveness of group therapy and the possibility of their progress.	1. Share examples of positive changes made by other members of the group (e.g., ‘A previous member overcame social anxiety through the group’). 2. Guide members to focus on small improvements in themselves or others (e.g., ‘You just took the initiative to express your feelings, which was very brave’). 3. The therapist emphasises the long-term goals and milestones of the group (e.g., ‘We will gradually explore how to cope with stress in the coming weeks’).
Universality	Reduce members’ sense of loneliness and enhance their sense of belonging. Help members realize that “their problems are universal” and not personal flaws.	Structured exercises (e.g., ‘Take turns sharing something you think only you have experienced’ to discover commonalities). The therapist summarises members’ similar experiences (e.g., “Many people mentioned feeling unappreciated in the family”). Encourage members to respond to others with ‘me too’ to strengthen empathy.
Imparting information	Provide psychological knowledge and practical skills to enhance members’ cognitive coping abilities. Dispel misconceptions about problems and establish a scientific cognitive framework.	Psycho-educational Lectures (e.g., physiological mechanisms of anxiety, communication skills). 2. Sharing of coping strategies among members (e.g., ‘When I have insomnia, I use positive breathing’). 3. Recommended self-help resources (books, meditation apps).
Altruism	Enhance members’ self-worth and sense of efficacy by helping others. Break the passive role of being a “help-seeker” and promote equal interaction.	1. Design supportive tasks (e.g., ‘In pairs, take turns listening to each other’s concerns and giving feedback’). 2. Encourage members to give specific support (e.g., ‘Can you share how you handled a similar conflict?’). 3. Reinforce positive feedback on altruistic behaviors (e.g., ‘What you just suggested was very helpful to A’).
Corrective family recapitulation	Identify and correct maladaptive interaction patterns formed by members in their family of origin. Rebuild healthy interpersonal relationship experiences in a safe environment.	Explore family patterns that members repeat in groups (e.g., ‘Do you find that you react to B much like you do to your father?’). Guide members to try new behaviors (e.g., ‘Next time when C criticises you, try expressing feelings directly instead of being silent’). The therapist models non-judgmental responses as an alternative to denial or neglect in the family of origin.
Socializing techniques	Enhance members’ interpersonal sensitivity and communication skills. Reduce social avoidance or conflict behaviors and improve real-world adaptability.	1. Role-playing (e.g., mock interviews, refusing unreasonable requests). 2. Immediate feedback (e.g., ‘When you interrupted D, she looked frustrated.’). 3. Non-verbal communication training (e.g., eye contact, body language awareness).
Imitative behavior	Help members acquire adaptive behavioral patterns through observational learning. Reduce fear of “change” and provide role models for reference.	1. The therapist models healthy behaviors (e.g., self-disclosure of vulnerability: ‘I’ve been ashamed of my failures, too’). 2. Invite members who have made significant progress to share specific steps for change (e.g., ‘How I went from conflict avoidance to proactive communication’). 3. Guide members to observe and record how others have responded effectively.
Interpersonal learning	Reveal members’ blind spots in interpersonal patterns through group interactions. Promote awareness of the consequences of their own behaviors and encourage attempts to adjust.	Here-and-Now technique: focuses on group interactions in the present moment (e.g., ‘You just frowned at E. What happened?’). Feedback loop (e.g., members take turns describing their perceptions of a particular conflict). Therapist linking group interactions with members’ realistic relational patterns (e.g., “You are always worried about being excluded in the group, is this true in general?”).
Group cohesiveness	Establish a trusting and safe group atmosphere to facilitate deep engagement. Reduce defence mechanisms and enhance members’ emotional involvement.	1. Group contract development (principle of confidentiality, commitment to participation). 2. Ice-breaking activities (e.g., ‘Describe in three words how you feel at this moment.’). 3. Periodic review of progress in group relationships (e.g., ‘In the past two weeks, have you felt safer in the group?’).
Catharsis	Release suppressed emotions and alleviate psychological stress. Promote self-acceptance and cognitive integration through emotional expression.	1. Guiding members to express emotions through physical movement or art (e.g., drawing, empty chair techniques). 2. Supporting emotional outbursts in a safe environment (e.g., allowing crying, keeping the group company rather than interrupting). 3. Guiding cognitive reframing after catharsis (e.g., ‘Is there an unmet need hidden behind the anger?’).
Existential factors	Help members confront fundamental life issues (such as loneliness, freedom, and death). Facilitate exploration of personal responsibility and the meaning of life.	1. Philosophical discussions (e.g., ‘How to face uncertainty in life?’). 2. Philosophical discussions (e.g., ‘How to face uncertainty in life?’) 2. Guiding members to reflect on their personal values (e.g., ‘What would you choose if you had only one year left to live?’). 3. Explore existential meaning through the sharing of themes of loss (e.g., loss of a beloved object, departure of a loved one).

In the first unit, in addition to establishing group norms and signing confidentiality agreements, the primary focus is sharing positive change cases from other group members to guide participants in noticing their own or others’ minor progress. This helps members recognize the effectiveness of group therapy and the possibility of their improvement. “Universality and Imparting Information” is the theme of the second unit, where members take turns sharing experiences they once believed were unique to themselves, discovering commonalities, and engaging in psychological lectures to encourage active sharing of coping strategies. This fosters mutual familiarity and recognition among members, creating an accepting and harmonious group atmosphere. It helps participants feel psychologically safe and relaxed within the group, realizing that “their issues are universal” rather than personal flaws. The psychoeducational lectures also provide psychological knowledge and practical skills, enhancing members’ cognitive coping abilities, dispelling misconceptions about their problems, and establishing a scientific cognitive framework. The third unit covers the fourth to fifth factors, focusing on targeted training for adolescents’ cognitive limitations, social skills, and dysfunctional family interaction patterns, improving members’ interpersonal sensitivity and communication abilities while strengthening their real-world adaptability. “Socializing Techniques” is the fourth unit, which uses activities such as mock interviews and refusing unreasonable requests to enhance members’ interpersonal sensitivity and communication skills, reducing social avoidance or conflict behaviors and improving real-world adaptability.

The fifth unit (seventh to eighth factors) reduces fear of “change” through behavioral modeling and interpersonal learning, provides reference role models, promotes awareness of the consequences of their actions, and encourages attempts at adjustment. The sixth unit (ninth factor) establishes a trusting and safe atmosphere among group members, fostering deep engagement, reducing defensive mechanisms, and strengthening emotional involvement. The seventh unit (10th factor) effectively guides the release of suppressed emotions to alleviate psychological stress, promoting self-acceptance and cognitive integration through emotional expression. The eighth unit (11th factor) explores profound philosophical questions to help members confront essential life issues, encouraging reflection on personal responsibility and the meaning of life. Each patient group undergoes two professional supervision sessions within the cycle to ensure therapeutic effectiveness.

Furthermore, during the group psychotherapy process, the therapist fully considers the interrelatedness of the factors in Yalom’s theory. In the early stages, the focus is on “instillation of hope” and “universality.” During the middle phase, the emphasis shifts to strengthening “interpersonal learning” and “socializing techniques.” In the later stages, the focus is on integrating “existential factors,” flexibly guiding the interaction between these factors (e.g., combining “catharsis” with “imparting information” for cognitive education).

(2) Control Group: Patients in the control group received medication with SSRIs (fluoxetine or escitalopram) starting at the lowest dose (fluoxetine 10 mg/day; escitalopram 5 mg/day). The dose was adjusted weekly according to clinical response and tolerability, not exceeding the maximum dose approved by the State Drug Administration (fluoxetine 40 mg/day; escitalopram 20 mg/day) or the patient’s tolerance limit. The medication treatment will continue for 4 weeks.

### 2.4 Observation indicators

#### 2.4.1 Depression status

We used the observer-rated scale, namely the 17-item Hamilton Depression Rating Scale (HAMD-17), to assess the depressive symptoms of the patients. The scale was assessed by a professional psychiatrist based on the patient’s condition in the last 2 weeks and consisted of 17 items. The items were rated on a scale of 0–2 or 0–4, with a total score ranging from 0 to 52. A score of 0–7 is no depression, 8–16 is mild depression, 17–23 is moderate depression, and 24 and above is severe depression. The higher the score, the more severe the depression. The scale consists of five factors: retardation, anxiety somatisation, cognitive impairment, sleep disorder, and weight.

#### 2.4.2 Anxiety

We used the Hamilton Anxiety Scale (Wu, 2009) to assess. The scale was assessed by a professional psychiatrist based on the subject’s situation in the last 2 weeks and consisted of 14 items, all of which were rated on a 5-point scale from 0 to 4. It contains two factors, psychogenic and somatic anxiety. Scale scores ranged from 0 to 56, with 17 and below indicating no anxiety, 18–25 indicating mild anxiety, 25–30 indicating moderate anxiety, and 30 or more indicating severe anxiety.

#### 2.4.3 Score reduction rate

HAMD/HAMA score reduction rate = (Total Score Before Intervention − Total Score After Intervention)/Total Score Before Intervention × 100%. A post-treatment HAMD/HAMA score reduction rate of ≥50% is generally considered to be clinically effective.

### 2.5 Statistical methods

Statistical software is SPSS 22.0, the individual normal data will be processed, represented by x ± s, the results between the groups will be tested by *t*-test, the results presented in cases (%) will be the count data contrast value, between the groups will be tested by *χ*^2^ test, if *p* < 0.05, then it means that the comparison results are statistically significant. ANCOVA was used for *post-hoc* analysis of HAMA outcomes to control for baseline differences.

## 3 Results

### 3.1 Comparison of HAMD-17 indicators between groups before and after intervention

There is no significant difference in the comparison of HAMD-17 indicators before intervention (*p* > 0.05); after symptomatic treatment for patients, the HAMD-17 score of the observation group is lower than that of the control group, and there is a significant difference in the comparison of scores at different stages within the group (*p* < 0.001), see Table 3.

**Table 3:** Comparison of HAMD-17 indicators between groups before and after intervention (x ± s).

Groups	Number of examples	Pre-intervention	Post-intervention	*Z*-value	*p*-value
Observation group	46	15.74 ± 0.85	7.02 ± 0.75	−8.418	<0.001
Control group	46	15.85 ± 0.79	8.50 ± 0.98	−8.375	<0.001
*Z*-value		−0.455	−6.398		
*p*-value		0.649	<0.001		

### 3.2 Comparison of HAMA indicators between groups before and after intervention

HAMA indexes have differences before intervention (*p* < 0.05); after symptomatic treatment for patients, the HAMA score of the observation group is lower than that of the control group, and there is a significant difference in the comparison of scores at different stages within the group (*p* < 0.001), see Table 4.

**Table 4:** Comparison of HAMA Indicators between groups before and after intervention (x ± s).

Groups	Number of examples	Pre-intervention	Post-intervention	*Z*-value	*p*-value
Observation group	46	15.17 ± 1.12	5.70 ± 0.84	−8.380	<0.001
Control group	46	15.63 ± 1.02	6.96 ± 1.11	−8.351	<0.001
Z-value		−2.120	−5.181		
*p*-value		0.034	<0.001		

### 3.3 Comparison of HAMD-17 reduction rates between groups

Comparison of HAMD-17 effective rate, the observation group was higher than the control group (*p* < 0.05), see Table 5.

**Table 5:** Intergroup comparison of HAMD-17 score reduction rates [*n* (%)].

Groups	Number of examples	Effective	Non-effective	Total effective rates
Observation group	46	41	5	89.1%
Control group	46	16	30	34.8%
Chi-square value				28.822
*p*-value				<0.001

### 3.4 Comparison of HAMA reduction rates between groups

Comparison of HAMA effective rate, the observation group was higher than the control group (*p* < 0.05), see Table 6.

**Table 6:** Intergroup comparison of HAMA score reduction rates [*n* (%)].

Groups	Number of examples	Effective	Non-effective	Total effective rates
Observation group	46	45	1	97.8%
Control group	46	37	9	80.4%
Chi-square value				7.180
*p*-value				0.007

### 3.5 Comparison of drug adherence indicators between groups before and after intervention

There is no significant difference in drug adherence indexes compared before the intervention (*p* > 0.05); after treating the patients, the drug adherence indexes of the observation group are higher than those of the control group, and the difference between the pre- and post-intervention comparisons of each group is significant (*p* < 0.05), see Table 7.

**Table 7:** Comparison of medication adherence indicators between groups before and after intervention (x ± s).

Groups	Number of examples	Pre-intervention	Post-intervention	*Z*-value	*p*-value
Observation group	46	3.32 ± 1.10	7.77 ± 0.19	−8.396	<0.001
Control group	46	3.34 ± 0.91	7.50 ± 0.72	−8.318	<0.001
*Z*-value		−0.192	−1.813		
*p*-value		0.848	0.070		

### 3.6 Comparison of HAMD-17 dimensions between groups before and after intervention

Anxiety somatisation items were investigated before the intervention, and the difference in data comparison was not significant (*p* > 0.05); after the intervention, the indicators of anxiety somatisation in the observation group were lower than those in the control group, and the difference in data comparison between the two groups before and after the intervention was significant (*p* < 0.05), see Table 8.

**Table 8:** Pre-intervention and Post-intervention comparisons between groups of HAMD-17 dimensions (x ± s).

Item	Time	Observation group	Control group	*Z*-value	*p*-value
Retardation	Pre-intervention	4.63 ± 0.74	4.33 ± 0.82	−2.030	0.042
	Post-intervention	3.93 ± 0.70	3.46 ± 0.78	−2.950	0.003
	*Z*-value	−4.255	−4.608		
	*p*-value	<0.001	<0.001		
Anxiety somatisation	Pre-intervention	10.13 ± 0.98	10.39 ± 0.91	−1.202	0.229
	Post-intervention	3.09 ± 0.66	4.72 ± 0.83	−7.412	<0.001
	*Z*-value	−8.426	−8.392		
	*p*-value	<0.001	<0.001		
Sleep disorders	Pre-intervention	3.22 ± 0.66	2.74 ± 0.80	−2.915	0.004
	Post-intervention	0.57 ± 0.65	0.07 ± 0.25	−4.459	<0.001
	*Z*-value	−8.378	−8.774		
	*p*-value	<0.001	<0.001		
Weight	Pre-intervention	0.98 ± 0.33	1.13 ± 0.34	−2.109	0.035
	Post-intervention	0.00 ± 0.00	0.33 ± 0.47	−4.210	<0.001
	*Z*-value	−8.856	−6.844		
	*p*-value	<0.001	<0.001		

### 3.7 Comparison of HAMA dimensions between groups before and after intervention

After the intervention, the scores of each item in the observation group were lower than those in the control group, and the difference between the data of the two groups before and after the intervention was significant (*p* < 0.05), see Table 9.

**Table 9:** Comparison of HAMA dimensions between groups before and after intervention (x ± s).

Item	Time	Observation group	Control group	*Z*-value	*p*-value
Somatic anxiety factor	Pre-intervention	4.98 ± 1.02	6.39 ± 0.88	−5.874	<0.001
	Post-intervention	2.02 ± 0.75	2.74 ± 0.74	−4.283	<0.001
	*Z*-value	−8.258	−8.414		
	*p*-value	<0.001	<0.001		
Mental anxiety factor	Pre-intervention	10.20 ± 0.88	9.24 ± 1.04	−4.558	<0.001
	Post-intervention	3.67 ± 0.99	4.22 ± 1.13	−2.151	0.031
	*Z*-value	−8.360	−8.346		
	*p*-value	<0.001	<0.001		

## 4 Discussion

### 4.1 Key findings

This study employed a randomized controlled trial to evaluate the clinical efficacy of group psychotherapy combined with antidepressant medication in patients with depression. Through a continuously revised and improved group psychotherapy protocol, the observation group demonstrated superior therapeutic effects on depressive and anxiety symptoms compared to the control group at the end of the 4th week of treatment. After 4 weeks of intervention, both groups showed significant reductions in depression and anxiety scores. Notably, patients receiving combined group psychotherapy and antidepressant medication exhibited significantly lower total depression scores, total anxiety scores, and somatic anxiety factor scores compared to the medication-only control group. The findings indicate that antidepressant medication effectively alleviates both depressive and anxiety symptoms in patients with depression, while the combination of antidepressants with group psychotherapy demonstrates enhanced therapeutic effects on emotional symptoms compared to pharmacotherapy alone, particularly showing greater efficacy in anxiety improvement. These results align with the majority of current research findings (Beck and Coffey, 2005; Hanieh et al., 2023; Jade and John, 2024).

In this study, group psychotherapy demonstrated significant improvements in depressive symptoms and cognitive function, effectively enhancing clinical treatment outcomes and shortening the treatment duration. The group psychotherapy model follows a fixed structure and methodology, offering strong operability and a short treatment cycle. Each session adheres to a structured process, ensuring high efficiency while alleviating patients’ anxiety and confusion. Through each round of interpersonal interaction, every patient is actively engaged, stimulating their autonomy and initiative, which highlights the advantages of group therapy (Yang and Song, 2025). Notably, the combined therapy group demonstrated superior medication adherence vs. controls (Table 6). We attribute this to: group-driven trust in treatment efficacy through observed peer progress; psychoeducation demystifying medications and accountability structures via family involvement and peer modeling. By mitigating stigma and misinformation—common adherence barriers in adolescents—group therapy created a self-reinforcing ecosystem supporting pharmacological engagement (Bertelsen et al., 2022). This approach shifts away from the traditional passive patient reception model, enabling individuals to adjust and improve their relationships with others, thereby fostering better social adaptation skills. This finding aligns with the conclusions of Yu Rui’s research (Du, 2015), which emphasizes that human lifestyles are inherently intertwined with group dynamics.

Additionally, this treatment method helps improve the quality of life for adolescent depression patients, enhances their treatment compliance, and facilitates their reintegration into school and society (Xu et al., 2011). Group therapy simulates a real social environment, providing patients with a trusting and supportive atmosphere (Yang et al., 2024; Vernmark et al., 2010). In this setting, members gain deeper self-awareness through observation, learning, experience, and interaction, while receiving encouragement and support from others. This process boosts their confidence, reduces avoidance behaviors, and ultimately alleviates depressive symptoms. It helps adolescent patients reintegrate into school and society, restoring their ability to learn and live normally. The role of group psychotherapy in improving social functioning and quality of life for adolescent depression patients surpasses that of individual therapy or medication alone (Shi and Luo, 2011; Yu et al., 2012). Furthermore, the universality and cohesion of the group significantly reduce the sense of stigma among adolescent depression patients, enhance their self-efficacy, and help restore their self-esteem, enabling them to confront their illness and effectively alleviate depressive and anxiety symptoms. Critically, these factors operated interactively: Universality reduced stigma, enabling honest engagement in Interpersonal Learning; Altruism reinforced self-efficacy, amplifying Imitative Behavior. This synergy—impossible in individual therapy—explains accelerated recovery in combined treatment, particularly for anxiety-driven avoidance (HAMA reduction: 97.8% vs. 80.4%, *p* = 0.007).

The reasons for patient dropout in this study are as follows: (1) Insufficient understanding of group psychotherapy among members, leading to a decline in motivation to seek psychological treatment during the waiting period; (2) Group psychotherapists may find it challenging to provide equal attention or support to each member during the treatment process, which could trigger significant emotional fluctuations in depression patients and result in dropout; (3) Some patients experience a high level of stigma, fearing they might encounter someone they know in the group or that group members may not adhere to the principle of confidentiality. Therefore, future research could consider increasing public awareness of mental illnesses and psychotherapy, as well as enhancing the professional skills of group leaders. Although we have a relatively low dropout rate (8%), attrition stems primarily from misunderstandings of treatment concepts, perceived unequal therapist attention, and concerns about stigma. We mitigate these issues through psychoeducation, co-therapy models, and confidentiality agreements.

### 4.2 Strengths

This study adopted a randomised controlled trial design and used computer-generated random allocation (1:1 allocation) to ensure minimal selection bias. At the same time, the first randomised controlled trial to systematically apply the 11 therapeutic factors of the A-Lon system to a Chinese adolescent population was conducted, and an eight-session structured group intervention programme was designed. The treatment demonstrated excellent retention rates, validating its feasibility in real-world settings.

### 4.3 Cultural implications

In China’s collectivist society, group psychotherapy leverages cultural values of community and shared responsibility. The observed efficacy of ‘universality’ and ‘altruism’ factors (Table 2) may reflect adolescents’ response to peer recognition in a culture that emphasises social harmony. Family participation aligns with Confucian familism and may enhance treatment acceptance (Bai, 2021). However, dropout cases associated with stigma (four cases) indicate cultural barriers that require community anti-stigma programmes. Future studies should examine how cultural factors moderate therapeutic mechanisms.

## 5 Limitation

This study also has certain limitations. Firstly, it is not a multicenter randomized controlled trial, and the single source of patients may introduce selection bias. Therefore, future multicenter randomized controlled trials are needed to further validate the effectiveness of group psychotherapy for depression. Secondly, the sample size of this study is relatively small and limited to specific regions or institutions, which may restrict the representativeness and generalizability of the findings. Thirdly, this study did not conduct follow-up assessments after the 4-week observation period for both the intervention and control groups, making it impossible to determine the long-term efficacy of group psychotherapy. Despite these limitations, this study provides preliminary evidence for the application of group psychotherapy in the treatment of adolescent depression. Future research should address these limitations by incorporating neuroimaging or biomarker detection to quantify the impact of group psychotherapy on neuroplasticity. While our findings demonstrate acute-phase advantages for combined therapy, the critical question—‘Do these gains translate to lasting recovery?’—remains unanswered. Future work must adopt developmentally sensitive longitudinal designs to evaluate whether early symptom improvement catalyzes resilient adulthood trajectories. Fourthly, due to the inherent visibility of group psychotherapy, neither participants nor therapists were able to remain blind to group assignment. There is a risk of performance and detection bias with this open-label design, although we mitigated this by using blinded outcome assessors and a standardised medication regimen. Future trials may partially address this limitation by using an active control groups to partially address this limitation. Limitations include baseline HAMA imbalance, though its small magnitude and ANCOVA-adjusted results reinforce our conclusions. Fifthly, despite randomization, a baseline imbalance in HAMA scores (*p* = 0.034) was observed. Although the absolute difference was small and both groups remained within the same clinical severity band (mild anxiety), we did not statistically adjust for this difference using ANCOVA in our primary analysis. This represents a methodological limitation that may influence the interpretation of HAMA outcomes. Additionally, further investigation is needed to test the generalizability of the treatment protocol across adolescents from different cultural backgrounds. The development of digital group intervention formats could also enhance accessibility. These steps will help further validate the efficacy of group psychotherapy and provide more robust scientific evidence for its application in clinical practice. In summary, the preliminary findings validate that group psychotherapy combined with medication has achieved significant clinical efficacy in the short term for treating adolescent depression, demonstrating a certain synergistic or enhancing effect. This combined approach effectively alleviates depressive and anxiety symptoms in patients.

## Data Availability

The raw data supporting the conclusions of this article will be made available by the authors, without undue reservation.
